# Hospital Meetings

**Published:** 1895-07-06

**Authors:** 


### HOSPITAL MEETINGS.

### THE GRAVESEND HOSPITAL.

#### Opening of New Wing.

On Tuesday, June 25th, the newly-built wing of the Gravesend Hospital was formally opened by the Mayor, Mr. E. C. Paine. The weather was brilliant and the inhabitants of Gravesend did their duty nobly in the way of bunting. The Mayor and Corporation walked in procession to the hospital, where they were welcomed by the chairman of the committee shortly after three o'clock. After inspecting the new building a move was made to a marquee in the grounds, where by this time many other friends of the hospital had collected, and here, after speeches from the chairman and Mr. C. E. Hatten (the hon. treasurer), a silver key was presented to the Mayor by the chairman, and the new wards having been declared open special prayers were said by the rector of Gravesend, the Rev. J. H. Haslam.

Afterwards purses were presented to the Mayoress by a number of children, the total amount thus given and promised being £248 8s. 10d. Other speeches followed, in which hearty testimony was borne to the generous help of many friends, more especially perhaps from Mr. Tingey and Mr. Paine, towards the hospital's needs. Owing to the action of the latter the whole of the proceeds of the forthcoming regatta would be devoted to its use, and the Treasurer called upon those present to remember that in spite of present support the ordinary income of the hospital had suffered from the effort to augment the building fund during the past year, and that the increased accommodation naturally meant increased expenditure, consequently requiring additional funds.

Refreshments were presently served to the guests, who spent the rest of the afternoon going over the new wards.

The new wing, of which the architect is Mr. Norman Nisbett, contains on the ground and first floors two wards each, one for 12 beds and a smaller one for six beds. The wards on the ground floor are intended for men, those above for women, and there is also on each floor a small room which may be used for special cases. Every modern improvement in the way of fireplaces, windows, rounded corners, heating appatatus, &c., has been introduced, and by further alteration in the older part of the hospital very much more convenient quarters are now provided for the nursing staff.

### THE CHELSEA HOSPITAL FOR WOMEN.

The report of the council of this hospital for the year ending December 31st, 1894, which was submitted at the annual meeting of the governors on June 26th makes reference to "the time of trial and trouble" through which the hospital has recently passed. The hospital, it is stated, "has emerged from the fire of criticism purified and strengthened." It was closed for the first four months of the year for the purpose of putting it in a thoroughly satisfactory sanitary condition. These improvements have been carried out at a cost of £1,200, "and the committee have the assurance of knowing that the sanitary arrangements are now beyond all suspicion." The committee refer with gratitude to the tokens of sympathy and support they have received from members of the Royal Family, and from the governors and subscribers. Legacies fell off greatly in 1894, only £200 having been received under this head; but in the present year (1895) legacies amounting to £2,500 have come in. The council deeply regret to record the death of the late Mr. Henry Whiting, a staunch friend of the hospital. The report is supplemented by tables which show in detail the results of treatment at the hospital for the ten months following the appointment of the new staff. As to expenditure, it is pointed out that the cost of the new drainage system and the fact that the hospital was closed for a third of the year have greatly increased the average cost per bed for the twelve months under notice. Moreover, the council point out that the number of patients at that hospital requiring most skilful operation and subsequent expensive dressings and diet, is abnormally large.

Sir ALGERNON BORTHWICK, M.P., who presided, moved the adoption of the report. He was glad to say that the governors were in a position to congratulate themselves. The Council had complied with every requirement of the special committee of inquiry of which Lord Balfour of Burleigh was chairman. The board of management had been reconstituted, and they had enlisted the services of an admirable medical staff. (Hear, hear.) He drew special attention to the statistics as to the result of treatment and the result of operations at the hospital, which showed a mortality of 1·8, as low a rate of mortality as had ever been presented. (Hear, hear.) He was glad to know that the governors and the public understood how good the work done at that hospital was, and that the funds were keeping up. They had begun the year well; they had had Royal favour, and they had had numerous subscriptions. Old memories had died down and they could now go forward with their work with confidence.

Colonel the Hon. W. J. COLVILLE, C.B., seconded.

A GOVERNOR asked if it was the case that the number of operations which had gone down for a time had lately gone up? Why had the name of the Princess of Wales ward been changed to that of another member of the Royal Family?

The CHAIRMAN replied that no operations had taken place without the best medical advice; that the Alexandra ward was now known as the Alexandra floor.

The report was then adopted.

Mr. BEADON moved the confirmation of the appointments of the medical staff, which had been made provisionally.

This was seconded and agreed to.

Asked why the consulting staff had not been appointed, the CHAIRMAN stated that he had approached several leading physicans and surgeons on the subject, but it was thought best to wait in the meantime. Delicate points had arisen, and the heads of the profession were chary about joining them in the meantime. By and by he had no doubt they would come in. The Chairman also pointed out that all the gentlemen who had been appointed to the medical staff had been approved of by the advisory committee.

A cordial vote of thanks was passed to Sir Algernon Borthwick, whose services to the hospital were warmly eulogised.

---

### NORTH LONDON HOSPITAL FOR CONSUMPTION.

ON Wednesday, June 26th, Mr. Noel E. Buxton took the chair at the festival dinner in aid of the funds of this hospital. The guests were many, including Sir T. F. Buxton, Lord Kinnaird, the Japanese Minister, Sir J. H. Kennaway, Dr. F. Winslow, and numerous other friends of the charity.

The JAPANESE MINISTER proposed the toast of "The Rulers of the British Empire," and commented in his speech upon the one thing which especially struck him in this country, the great attention paid to charity. It was, he thought, one of England's characteristics, and it was gratifying to find that there was hardly an English man or woman of means who did not contribute his or her share to that great cause.

Lord KINNAIRD, Sir JOHN KENNAWAY, and Sir T. F. BUXTON responded, and then followed the toast of "Prosperity to the North London Hospital for Consumption." The CHAIRMAN, in a happy speech, said that before accepting the invitation to take the chair he had been anxious to satisfy himself that the object was a good one, that a dinner was essential, and that he himself was the proper person to preside. On the first of these points a personal visit to the hospital had assured him that it was the best of its kind, if only on account of its excellent position, and the secretary persuaded him as to the second, while on the third he was relieved to find an amusing speech or a passionate appeal was not expected, but, what he would try to give them, one which should be business-like. Several friends, said Mr. Buxton, in sending their cheques, had protested that hospitals ought to be paid for out of the rates, and he agreed with them that hospitals should be subsidised with public money, though still managed by those who gave voluntary support. It was needless to touch upon the well-known and terrible ravages of consumption in this country. In this there was a melancholy bond of sympathy between England and Japan, where the percentage of deaths from that disease was even higher than with us. With regard to the hospital itself, it had been founded in 1860, was soon found to be too small, and in 1880 a new building was begun, of which the central block and one wing were finished. The annual income was insufficient to thoroughly equip the wards, and a small increase in receipts would mean a large increase in the work done. The cost of each patient was high, compared with that at the hospital he knew best, the London, but it was to be remembered that the patients often were suffering from bad food, and only the best was, therefore, necessary. Touching upon the Christian side of the work, Mr. Buxton said he should have had little pleasure in taking the chair that night were it not that the committee thought so much of this question that they went to some private expense to provide a chaplain, and he rejoiced to think that whether in sadness or rejoicing trouble was taken to put before each inmate the only permanent comfort anyone could know. In conclusion, the case was, he thought, a strong one, and in the face of so much distress a few paltry guineas should not be grudged which would mean so little to the givers, so much to those who benefited thereby.

Subscriptions were afterwards announced amounting to something over £1,700, and other toasts followed, while the music of the Westminster Singers and Miss Mabel Chaplin's 'cello solos were much appreciated by all present.

